# Correction: BOADICEA: a comprehensive breast cancer risk prediction model incorporating genetic and nongenetic risk factors

**DOI:** 10.1038/s41436-019-0459-4

**Published:** 2019-02-21

**Authors:** Andrew Lee, Nasim Mavaddat, Amber N. Wilcox, Alex P. Cunningham, Tim Carver, Simon Hartley, Chantal Babb de Villiers, Angel Izquierdo, Jacques Simard, Marjanka K. Schmidt, Fiona M. Walter, Nilanjan Chatterjee, Montserrat Garcia-Closas, Marc Tischkowitz, Paul Pharoah, Douglas F. Easton, Antonis C. Antoniou

**Affiliations:** 1 Centre for Cancer Genetic Epidemiology, Department of Public Health and Primary Care, The Strangeways Research Laboratory, University of Cambridge, Cambridge, UK; 2 Division of Cancer Epidemiology and Genetics, National Cancer Institute, National Institutes of Health, Rockville, MD USA; 3 The Primary Care Unit, Department of Public Health & Primary Care, University of Cambridge, Cambridge, UK; 4 Hereditary Cancer Program, Epidemiology Unit and Girona Cancer Registry, Catalan Institute of Oncology, Girona Biomedical Research Institute (IdiBGi), Girona, Spain; 5 Centre Hospitalier Universitaire de Québec–Université Laval Research Center, Québec City, QC Canada; 6 Division of Molecular Pathology, Netherlands Cancer Institute, Amsterdam, The Netherlands; 7 Department of Biostatistics, Bloomberg School of Public Health, Johns Hopkins University, Baltimore, MD USA; 8 Department of Oncology, School of Medicine, Johns Hopkins University, Baltimore, MD USA; 9 Department of Medical Genetics and National Institute for Health Research, Cambridge Biomedical Research Centre, University of Cambridge, Cambridge, UK; 10 Centre for Cancer Genetic Epidemiology, Department of Oncology, University of Cambridge, Cambridge, UK

Correction to: *Genetics in Medicine* 10.1038/s41436-018-0406-9; published online 15 January 2019

The original version of this article contained a typographical error in Eq. 2. The summation in the equation should run up to $\mu = 6$, and so Eq. 2 should have read

$$\lambda ^{\left( i \right)}\left( t \right) = \lambda _0\left( t \right){\mathrm{exp}}\left( {\mathop {\sum }\limits_{\mu = 1}^6 \left( {\beta _{MG\mu }\left( t \right) + \mathop {\sum }\limits_\rho {\boldsymbol{\beta }}_{RF\rho \mu }\left( t \right) \cdot {\boldsymbol{z}}_{RF\rho }^{\left( i \right)}} \right)\mathop {\prod }\limits_{\nu = 1}^{\mu - 1} \left( {1 - G_\nu ^{\left( i \right)}} \right)G_\mu ^{\left( i \right)} + \beta _{PG}\left( t \right)x_P^{\left( i \right)}} \right),$$

where $\mu = 6$ corresponds to a noncarrier of pathogenic variants in the major genes, with $\beta _{MG6}\left( t \right) = 0$, and $G_6^{\left( i \right)} = 1$ for noncarriers of pathogenic variants, and 0 otherwise. All presented results and conclusions were based on the correct version of the equation and are not influenced by this typographical error.

This has now been corrected in both the PDF and HTML versions of the Article. The authors regret this error.

